# Peripheral blood RNA-seq analysis in bipolar and schizophrenia spectrum disorders: modest influence of antipsychotic treatment

**DOI:** 10.3389/fphar.2026.1745052

**Published:** 2026-02-18

**Authors:** Yosra Bejaoui, Suhaila Ghuloum, Teena John, Gaurav Thareja, Tanwir Habib, Karsten Suhre, Hassen Al-Amin

**Affiliations:** 1 Psychiatry Department, Weill Cornell Medicine in Qatar, Doha, Qatar; 2 Mental Health Services, Hamad Medical Corporation, Doha, Qatar; 3 Bioinformatics Core, Research Department. Weill Cornell Medicine in Qatar, Doha, Qatar

**Keywords:** antipsychotics, bipolar disorder, RNA seq, schizophrenia spectrum disorders, Transcriptomics

## Abstract

**Introduction:**

Bipolar disorder (BP) and schizophrenia spectrum disorders (SSD) are chronic mental health conditions that share genetic risks. Despite extensive research, the biological mechanisms underlying these disorders are still not fully understood.

**Methods:**

In this study, we analyzed peripheral blood RNA-seq data from 64 patients with BP and SSD, both on and off antipsychotic treatment, and 92 healthy controls. We conducted differential gene expression analyses, pathway enrichment analyses, weighted gene co-expression network analysis (WGCNA), and clinical trait association analyses.

**Results:**

Patients showed distinct blood transcriptomic profiles compared to controls, with a core set of 1,278 differentially expressed genes shared between individuals with mental illness, both on and off antipsychotics. These common DEGs were enriched in immune processes, metabolic pathways, and cellular homeostasis functions. WGCNA identified a co-expression module that was strongly associated with mental illness status and not with metabolic traits. We also observed a significant group effect for total cholesterol, LDL cholesterol, systolic blood pressure, and diastolic blood pressure when comparing metabolic clinical features among MD, MD + APs, and HC.

**Conclusion:**

This transcriptomic study of BP and SSD in a Qatar-based cohort highlights dysregulated immune and metabolic pathways.

## 1 Introduction

Schizophrenia spectrum disorder (SSD) and bipolar disorder (BP) are chronic mental health conditions. They present as clinical disturbances in thinking, emotional regulation, and behavior, creating a significant burden on individual health and social life, and leading to premature mortality and morbidity (Arias et al., 2022). The reduced lifespan is attributed to both inherent genetic vulnerability and the effects of the antipsychotics used as treatment, which increase the risk of metabolic syndrome and cardiovascular disease (Popa et al., 2025; Chaudhari et al., 2025). These disorders may differ in symptoms, neuropsychology, and brain structure (Murray et al., 2004). Still, genetic evidence supports the idea that BP and SSD share molecular abnormalities, with genome-wide studies identifying common loci between the two disorders (Lichtenstein et al., 2009; International Schizophrenia et al., 2009). Their diagnoses rely on clinical symptoms, which results in high phenotypic heterogeneity.

The brain is the most relevant tissue for studying gene-expression abnormalities in psychiatric disorders; however, several studies have shown that blood gene-expression levels significantly correlate with those in the brain (McKenzie et al., 2014). Since brain tissue is inaccessible in living patients, peripheral blood has been widely used as a surrogate tissue for transcriptomic studies to identify biomarkers for mental illness. Blood-based transcriptomic analyses have demonstrated that BD and SSD exhibit distinct expression profiles capable of discriminating between diagnostic groups, while sharing alterations in immune and inflammatory pathways (Drexhage et al., 2010; Tsuang et al., 2005). Brain-based studies have identified schizophrenia-specific disruptions in metabolic and mitochondrial pathways, emphasizing tissue- and disorder-dependent molecular differences (Middleton et al., 2005). Hess et al. conducted a meta-analysis of whole-blood transcriptomes combining BP and SSD groups, revealing 13 altered gene modules enriched in apoptosis, oxidative stress, chromatin remodeling, and immune signaling (Hess et al., 2020).

One key factor to consider is how antipsychotic medications influence gene expression profiles in psychiatric disorders. Patients with SSD and BP are typically treated with different types of antipsychotic drugs, such as first-generation (FGA) or second-generation (SGA) APs, and some receive combined treatments. It is well documented that these medications can modify gene expression in peripheral blood (Wagh et al., 2021). Consequently, the widespread use of APs complicates differentiating between medication-induced expression changes and those inherent to the disorders (Schulmann et al., 2023). Furthermore, recent transcriptomic studies have shown that antipsychotic-induced metabolic changes likely originate from complex, multi-tissue gene network reprogramming, involving, for example, inflammatory cytokine signals in blood (Sun et al., 2024) and shared transcriptomic signatures of psychosis and blood glucose (Lee et al., 2024). These insights provide a molecular foundation for therapeutic targeting and early biomarker development in managing metabolic syndrome among individuals on antipsychotics.

Another gap in current research is the lack of diversity among the populations studied. Most blood-based gene expression studies in BP and SSD have been conducted in European, East Asian, or other non-Arab populations (Wagh et al., 2021). Addressing this bias is important, as more studies involving diverse populations will help establish reliable biomarkers. A previous study compared plasma proteomics profiles of schizophrenia and bipolar disorder patients, both on and off APs, to healthy controls in a Qatar-based population. It identified nervous system–related proteins (NTRK2, CNTN1, ROBO2, PLXNC1) that were downregulated in antipsychotic-free SDD and BP patients but appeared “normalized” in patients taking APs (Engelke et al., 2022).

In this study, we conducted a cross-disorder transcriptomic analysis of peripheral blood samples from a Qatar-based cohort, an underrepresented group in psychiatric research. The aim is to identify differentially expressed genes (DEGs) and shared pathogenic mechanisms in these disorders among patients on and off APs, compared to controls with no mental illness and not using any psychotropics. We also examined the relationships between the transcriptomic profile and antipsychotic-induced metabolic changes in this cohort.

## 2 Materials and methods

### 2.1 Study design and participants

This cross-sectional study is part of a project aimed at evaluating biomarkers in patients maintained on APs in Qatar. Refer to other published papers from this project for more details on the methodology (Hammoudeh et al., 2018; Engelke et al., 2022; Kiwan et al., 2019). The institutional review boards (IRB) at Hamad Medical Corporation (HMC; number 16-0002) and Weill Cornell Medicine in Qatar (WCMQ; number 1056791-10) approved the study. Patients (BP and SSD subjects) were recruited from Qatar’s Mental Health Services, and the control group was recruited from primary care clinics and visitors to the Mental Health Hospital. All participants provided written informed consent before enrollment. The presence or absence of a psychiatric disorder was confirmed using the standardized Mini International Neuropsychiatric Interview (Sheehan et al., 1998), which follows the Diagnostic and Statistical Manual (DSM) criteria for diagnosing psychiatric disorders.

Briefly, the inclusion criteria for patients were ages 18–65 years and at least 6 months of maintenance on APs. Patients in the MD + APs group were treated with various antipsychotic medications, including both FGA and SGA. Those not on APs met the same criteria, except they had been off APs for at least 6 months. We did not recruit first-episode subjects or participants completely naïve to psychotropics. The inclusion criteria for healthy controls (HC) included the same age range, but they had no mental illness or AP use. For this study, we selected a total of 64 patients diagnosed with SSD and BP, both on and off APs, along with 92 HC matched for age and gender.

### 2.2 Data and sample collection

Trained raters collected demographic and clinical data, including age, sex, and APs taken. They also measured body mass index (BMI), waist circumference, and blood pressure (systolic and diastolic). Fasting blood samples were collected (typically in the morning after 10–12 h of fasting) within 1–2 days of recruitment and stored for subsequent testing. Metabolic measures, including lipid profile (cholesterol, low-density lipoprotein (LDL), high-density lipoprotein (HDL), glycosylated hemoglobin, and fasting blood glucose, were measured directly from these samples using the same standardized procedures employed at Hamad General Hospital, Doha, Qatar.

### 2.3 Library construction sequencing

Paxgene Blood RNA (Qiagen Catalog no. 762164) kit was used to extract RNA from peripheral blood, and Qubit was used to determine the RNA concentration. To ensure high-quality RNA suitable for transcriptome profiling, rRNA and globin transcripts were depleted using Lexogen’s RiboCop rRNA Depletion Kit for Human/Mouse/Rat plus Globin. After depletion, stranded RNA-seq libraries were prepared using Lexogen’s CORALL RNA-Seq V2 Kit, which preserves strand orientation to enhance transcript resolution. All samples met the library quality control standards prior to sequencing. The libraries were then sequenced on the Illumina NovaSeq 6000 platform using the S4 v1.5 200-cycle configuration, producing high-throughput paired-end reads for downstream expression analysis. Sequencing summary statistics, including read depth and CG%, are provided in Supplementary Table S1. All samples contain over 61 million reads, with an average Q score of 35.

### 2.4 Data analysis

Transcript-level abundance estimates were obtained using the Illumina DRAGEN Bio-IT Platform, which generated quant. sf files for each sample *via* Salmon (Patro et al., 2017). The tximport R package was used to summarize transcript-level estimates into gene-level counts (Soneson et al., 2015), applying a transcript-to-gene mapping derived from the GENCODE v36 GTF annotation. These gene-level counts were then used to construct a DESeqDataSet object for differential expression analysis with DESeq2 (Love et al., 2014). Quality control steps included filtering out genes with counts <10, and no outlier samples were identified using hierarchical clustering and principal component analysis (PCA). Differential gene expression analysis was performed using the DESeq2 package (v1.46.0), with adjustment for gender and a significance threshold of an adjusted p-value (FDR) < 0.05 and a log2 fold change cutoff of 1.5. MA plots were generated using “lfcShrink” for all contrasts to assess dispersion patterns and potential biases caused by group size imbalance (Supplementary Figure S1). The PCA plot was generated using PCA Explorer (Bioconductor) based on variance-stabilized RNA-seq counts (VST) obtained from the DESeq2 pipeline after gene filtering.

Functional enrichment was then performed using the Metascape tool to identify specific altered pathways (Zhou et al., 2019). First, we compared mental disorder off APs (MD) and mental disorder on APs (MD + APs) *versus* HC to identify differentially expressed genes (DEGs). Subsequently, we categorized the groups by disease and compared each of the following groups: SSD, SSD + APs, BP, and BP + APs, independently vs. the HC. We then compared the identified DEGs with genes previously reported to be associated with SSD and BP in independent studies: (Pan et al., 2025; Wang et al., 2024; Cui et al., 2021; Hess et al., 2020; Krebs et al., 2020; Munkholm et al., 2015).

The demographic, clinical, and metabolic data were analyzed using SPSS (version 29), with ANOVA for continuous variables and Chi-Square tests for categorical variables to assess differences among the three groups. The p-value was set at 0.05, and Bonferroni corrections were applied to multiple *post hoc* comparisons, as specified in SPSS.

### 2.5 Weighted gene co-expression network analysis (WGCNA)

The main goal of network co-expression analysis is to identify biologically and clinically relevant modules and genes. A scale-free co-expression network was built using soft thresholding. The optimal soft threshold was selected to cluster this network into different functional modules. Modules were hierarchically clustered by calculating the eigengene, and similar modules were merged. Then, we ran a linear model to determine which modules contribute to differences across groups. We also correlated the modules identified through WGCNA with clinical metabolic features.

### 2.6 Protein–protein interaction (PPI) network analysis

Protein–protein interaction (PPI) network analysis was conducted using Metascape, an online platform (http://metascape.org). The list of differentially expressed genes (DEGs) was uploaded into Metascape for functional annotation and interaction analysis. PPI networks were created by integrating data from publicly available databases: STRING, BioGrid, OmniPath, and InWeb IM (Li et al., 2017; Stark et al., 2006; Szklarczyk et al., 2019).

## 3 Results

Our study cohort comprises three main groups: individuals with mental illness (MD) not receiving antipsychotic treatment (n = 29), individuals with mental illness on APs (MD + APs) (n = 35), and healthy controls (HC) with no mental illness or antipsychotic use (n = 92). Among those with mental illness, the off-antipsychotic group includes six individuals with SSD and 23 with BP, while the on-antipsychotic group includes 15 SSD + APs and 20 BP + APs patients (Table 1).

**TABLE 1:** Demographic and clinical characteristics by diagnosis.

Sample characteristics	HC	MD	MD + APs
Participants (n)	92	29 (BD:23; SSD: 6)	35 (BD: 20, SSD: 15)
Male/Female (n)	56/36	19/10 (BD:17/6, SSD:4/2)	25/10 (BD:14/6, SSD:11/4)
Age (yrs; M ± SD)	34.11 ± 9.13	34.61 ± 9.73	33.24 ± 11.60
Smoking history n (% within group)	15 (18%)^a^	10 (35%)	16 (46%)
Age onset of psychiatric symptoms (yrs; M ± SD)	​	28.48 ± 10.18	22.20 ± 8.25 ^b^
Age at first psychiatric diagnosis (yrs; M ± SD)	​	29.30 ± 9.45	22.61 ± 8.10 ^b^
Duration of illness (yrs; M ± SD)	​	5.88 ± 8.30	10.60 ± 10.10
APs (n)	​	​	FGA: 8, SGA: 20, both: 7

^a^ HC < MD + AP, b Age MD + AP < MD (p < 0.05). HC: healthy control; MD: mental disorder; APs: antipsychotics; BD: bipolar disorder; SSD: schizophrenia spectrum disorders; yrs: years; M ± SD: mean ± standard deviation; FGA: first-generation antipsychotics; SGA: second-generation antipsychotics. FGA, included chlorpromazine and haloperidol; SGA, included risperidone, olanzapine, quetiapine, aripiprazole, and clozapine.

**FIGURE 1:**
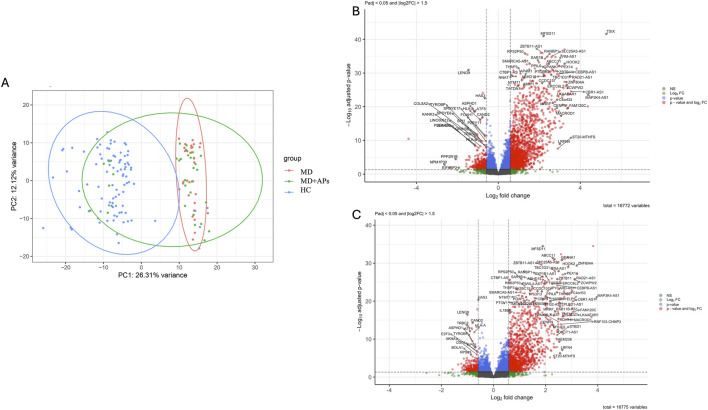
**(A)** Principal component analysis (PCA) of variance-stabilized RNA-seq data generated using PCA Explorer for HC, MD, and MD + APs study groups. **(B)** Differentially expressed genes between MD and HC (1701 upregulated, 453 downregulated) **(C)**: Differentially expressed genes between MD + APs and HC (1,354 upregulated and 255 downregulated). HC: Healthy controls, MD: mental illness off antipsychotics, MD + APs: mental illness on antipsychotics.

PCA was conducted to examine transcriptional variation across samples. Specifically, we analyzed samples categorized as HC, MD, and MD + APs. The first two principal components accounted for 38% of the total variance (PC1: 26.31%, PC2: 12.12%) (Figures 1–A). This revealed a separation between unaffected and affected individuals and showed that samples from individuals receiving antipsychotic treatment had greater transcriptional variability than those not on treatment. We then divided the samples based on mental illness diagnosis and antipsychotic treatment status into four groups. Overall, most affected samples were distinct from the healthy controls. However, a subset of MD + APs samples exhibited a partial shift toward the HC cluster, indicating variability within the treated group. (Supplementary Figure S2).

### 3.1 Differential gene expression in patients with mental illness, on and off antipsychotics

To explore the transcriptomic signatures in individuals with mental illness, we first performed a differential expression analysis comparing all individuals with mental illness to healthy controls (HC), regardless of antipsychotic treatment status. This analysis identified 1,565 upregulated genes and 276 downregulated genes (Supplementary Table S2). Functional enrichment analysis of the differentially expressed genes revealed pathways related to immune activation and immune cell differentiation, including positive regulation of immune response, T-cell activation, and selection. Additionally, enriched pathways involved RNA metabolism and translation, as well as DNA metabolic and repair processes. These findings suggest that mental illness is associated with dysregulation of immune, metabolic, and fundamental cellular regulatory processes in the peripheral blood (Supplementary Figure S3).

We then examined transcriptional differences between SSD and BP subjects, both on and off APs, and healthy controls. The analysis identified 2,154 and 1,611 genes that were differentially expressed in subjects off and on APs, respectively, compared to HC (Figures 1B,C) (Supplementary Tables S3-S4). We then compared subjects on *versus* off APs; this analysis identified only four genes that were differentially expressed, with one upregulated gene, CYRIA, and three downregulated genes, TSIX, FRS2, and XIST (Supplementary Table S5).

**FIGURE 2:**
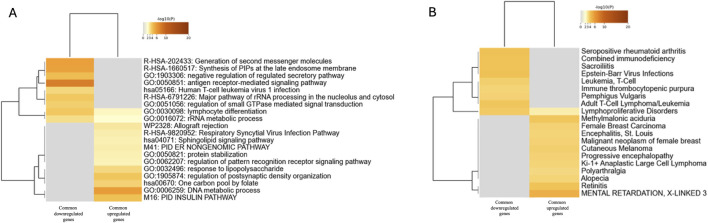
**(A)** Heatmap of enriched terms of common up- and downregulated genes between MD and MD + APs compared to HC, colored by p-values. **(B)** Summary of enrichment analysis in DisGeNET.

Venn diagram analysis revealed 1,189 common upregulated genes and 89 downregulated genes between the MD and MD + APs DEGs (Supplementary Figure S4A). Functional enrichment analysis was conducted using Metascape and represented in the heatmap (Figure 2A). We identified significant enrichment of immune- and infection-related pathways, such as antigen receptor-mediated signaling, regulation of pattern recognition receptor signaling, and lymphocyte differentiation, as well as processes related to RNA metabolism and protein regulation, including protein stabilization and postsynaptic density organization. Additionally, we observed enrichment in signaling and lipid metabolic pathways, including sphingolipid signaling, phosphatidylinositol phosphate synthesis, and metabolic processes such as the one-carbon folate pool, DNA metabolic process, and the PID insulin pathway. These findings suggest that both the MD and MD + APs groups share a core set of dysregulated pathways involving immune activation, RNA and protein homeostasis, and metabolic regulation. Disease enrichment analysis also revealed strong links to immune-related, infectious, and neoplastic disorders. The gene sets were associated with conditions such as cutaneous melanoma and female breast carcinoma and showed connections to neurological and metabolic conditions, including encephalitis and progressive encephalopathy (Figure 2B).

The enrichment analysis of protein-protein interactions (PPI) among the common DEGs is shown in Supplementary Figure S4B. The MCODE plugin, a tool for identifying functional modules in PPI networks, was used in this analysis. The top-scoring modules were “RHOD GTPase cycle,” “generation of second messenger molecules,” and “cytokine signaling in the immune system” (Supplementary Table S6).

To understand hidden patterns in the expression data and identify potential subgroups of genes with collective classification value, we performed WGCNA. We identified the module with the greatest differential expression across the groups (MD and MD + APs). This analysis revealed that module M1 has elevated expression in mentally ill subjects compared to healthy controls (P_adj_ = 8.017x10^−18^) (Supplementary Figure S5). Next, we checked whether the genes in M1 overlap with DEGs in MD and MD + APs compared with HC (Supplementary Table S7). We observed 966 common genes across the three datasets. These genes were enriched in immune-related pathways, such as lipopolysaccharide-mediated signaling and antigen receptor regulation, as well as metabolic and genomic processes, including folate metabolism, telomere maintenance, and ATR activation in response to replication stress. Disease enrichment further linked these genes to spinocerebellar ataxia, classical Hodgkin’s lymphoma, and acute coronary syndrome, indicating their relevance to both neuropsychiatric pathology and systemic comorbidities (Supplementary Figure S6).

### 3.2 Association of metabolic measures with mental illness in patients on and off antipsychotics

Our sample showed no significant differences in gender or age (Table 1). The smoking history showed a significant difference (Χ^2^ = 12.5, p = 0.002), and *post hoc* comparisons revealed that the proportion of smokers was significantly lower in the HC than in the MD + AP group (p < 0.05) (Table 1). The duration of mental illness was significantly different in the two patient groups, but the onset of symptoms and diagnosis was significantly earlier in the MD + APs than the MS off APs (p < 0.05) (Table 1).

**FIGURE 3:**
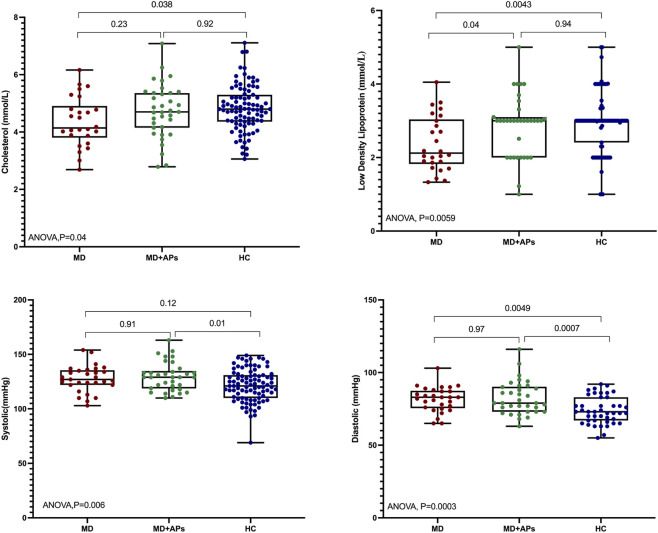
Metabolic features (Total cholesterol, LDL, systolic and diastolic blood pressure) in MD, MD + APs, and HC.

We compared a panel of metabolic clinical features among MD, MD + APs, and HC. BMI, waist circumference, fasting blood glucose, HbA1c, triglycerides, and HDL cholesterol did not differ significantly across the groups (Table 2; Supplementary Figure S7). In contrast, we observed significant group effects for LDL cholesterol (ANOVA*, p* = 0.001), systolic blood pressure (ANOVA*, p* = 0.007), and diastolic blood pressure (ANOVA*, p* = 0.004). Post-hoc comparisons (Table 2) revealed that LDL cholesterol levels were higher in MD + APs than in MD, while diastolic blood pressure was elevated in both MD and MD + APs compared to HC. Systolic blood pressure was only increased in MD + APs compared to HC (Figure 3). These findings suggest that cardiovascular risk markers, especially lipids and blood pressure, were altered in patients with or without antipsychotic treatment. We used WGCNA to explore the relationships between identified modules and clinical metabolic features. The module–trait heatmap revealed specific associations: ME126 was strongly correlated with waist circumference (*r* = 0.97, *q* = 1.4 × 10^−93^), while ME12 correlated with BMI (*r* = 0.36, *q* = 3.6 × 10^−4^), indicating gene clusters potentially linked to obesity-related pathways. Additionally, ME14 and ME71 both associated with HbA1c (*r* = 0.36, *q* = 2.4 × 10^−4^; *r* = 0.36, q = 3.5 × 10^−4^). ME32 correlated with fasting glucose (*r* = 0.46, *q* = 1.9 × 10^−7^), and ME122 was associated with triglyceride levels (*r* = 0.31, *q* = 5.2 × 10^−3^). HDL cholesterol showed correlations with ME138 (*r* = 0.51, *q* = 2.2 × 10^−9^) and ME89 (*r* = 0.33, *q* = 1.5 × 10^−3^). Blood pressure and pulse also demonstrated significant associations, with ME109 linked to systolic (*r* = 0.39, *q* = 4.9 × 10^−5^) and diastolic blood pressure (*r* = 0.38, *q* = 8.8 × 10^−5^). Overall, these results highlight co-expressed genes connected to metabolic and cardiovascular traits in our cohort (Supplementary Figure S8; Supplementary Table S8).

**TABLE 2:** Metabolic measures, ANOVA, and *post hoc P*-values of comparisons between MD, MD + AP, and HC.

*P-value*				
Metabolic measures	*ANOVA*	*MD - HC*	*MD + APs - HC*	*MD + APs - MD*
BMI	0.123	0.127	1.000	0.420
Waist circumference (cm)	0.310	1.000	0.638	0.448
Triglycerides (mmol/L)	0.902	1.000	1.000	1.000
HDL (mmol/L)	0.093	0.090	1.000	0.553
LDL (mmol/L)	0.001	<0.001	1.000	0.019
Systolic blood pressure (mmHg)	0.007	0.135	0.013	1.000
Diastolic blood pressure (mmHg)	0.004	0.040	0.011	1.000
Fasting glucose (mmol/L)	0.482	0.698	1.000	1.000

ANOVA: analysis of variance; MD: mental disorder; HC: healthy control; APs: antipsychotics. HDL: high-density lipoprotein; LDL: low-density lipoprotein.

### 3.3 Differential gene expression in bipolar and schizophrenia spectrum disorders on and off antipsychotics

**FIGURE 4:**
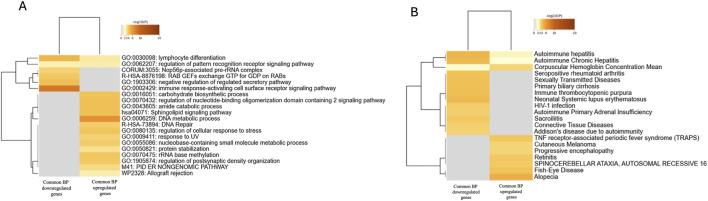
**(A)** Enrichment of common down and upregulated genes between BP off APs and BP on APs. **(B)** Summary of enrichment analysis in DisGeNET of common down and upregulated genes between BP off APs and BP on APs.

We then subcategorized the subjects with mental illness based on their diagnosis and compared the different groups. Comparing BP and BP + APs *versus* HC yielded 2,229 and 1936 DEGs, respectively (Supplementary Figure S9; Supplementary Tables S9-S10). A Venn diagram of the significant DEGs in both BP and BP + APs showed 1,273 shared upregulated genes and 100 downregulated genes (Supplementary Figure S10A). We performed functional enrichment analysis using Metascape, which is represented in the heatmaps (Figures 4A,B). This analysis revealed clusters of genes involved in immune-related and metabolic pathways. Notable terms included lymphocyte differentiation, regulation of antigen receptor-mediated signaling, negative regulation of the secretory pathway, sphingolipid signaling, and DNA repair. Disease enrichment analysis of the shared gene set indicated associations with various immune-mediated and systemic disorders, such as autoimmune hepatitis, primary biliary cirrhosis, systemic lupus erythematosus, connective tissue diseases, and HIV-1 infection. Additionally, enrichment in progressive encephalopathy, retinitis, and cutaneous melanoma suggests links to neurodegenerative and cancer-related pathways. MCODE clustering identified 11 functional modules, with key enrichments including small GTPase cycles and mitotic processes (MCODE1), purine and nucleotide metabolism (MCODE2), sphingolipid and ceramide metabolism (MCODE3), and snRNA processing (MCODE4) (Supplementary Figure S10B; Supplementary Table S11).

We then conducted differential expression analysis in SSD patients, both on and off APs, compared to HC, identifying 685 and 1,047 DEGs, respectively (Supplementary Tables S12, S13). A Venn diagram analysis showed 484 common upregulated and only seven common downregulated genes. Due to the small number of downregulated genes, enrichment analysis was performed only on the upregulated set. Functional annotation revealed significant enrichment in pathways related to *hexose catabolic processes, telomere maintenance in response to DNA damage, folate metabolism and transport,* r*RNA modification,* and *pyruvate metabolism.* Disease association analysis indicated enrichment in conditions such as *benign prostatic hyperplasia, spinocerebellar ataxia,* and *pancreatic ductal carcinoma*. These findings suggest that the transcriptomic changes common to SSD, independent of antipsychotic exposure, involve cellular metabolic pathways, genome maintenance, and immune-oncogenic processes (Supplementary Figure S11).

To assess the reproducibility of our results, we compared the differentially expressed genes (DEGs) identified in SSD patients (both on and off APs) with those reported in three transcriptomic studies (Pan et al., 2025; Wang et al., 2024; Cui et al., 2021). The overlap analysis showed that six genes (MATN1, PLIN4, DNAJB1, SLC22A4, CRYZ, and LIM2) were shared between our dataset and Pan et al., study (Pan et al., 2025), while another six genes (MFSD3, CSNK1D, MIB2, TMEM238, GPR152, and HRNR) overlapped with Wang et al., study (Wang et al., 2024). No genes overlapped with Cui et al., study (Cui et al., 2021). Notably, one gene, SPON2, was common across all our datasets and both studies by Pan et al. and Wang et al. (Pan et al., 2025; Wang et al., 2024). These overlapping genes may be robust candidates underlying the molecular pathology of SSD, irrespective of treatment status. We then compared the 1,376 DEGs common in BP patients on and off APs with transcriptomic signatures from three other studies. Only one gene, OGT, was shared between our dataset and Hess et al. study (Hess et al., 2020), with no overlapping genes observed with Krebs et al. or Munkholm et al. studies (Krebs et al., 2020; Munkholm et al., 2015). Each of these published studies showed different gene sets, highlighting the heterogeneity of blood-based transcriptomic profiles (Krebs et al., 2020; Munkholm et al., 2015) (Supplementary Figure S12).

## 4 Discussion

In our study, we examined global gene expression in 64 individuals with mental illness (29 off and 35 on antipsychotic medications) and 92 healthy controls. The observed transcriptional changes were derived from peripheral blood leukocytes and reflect systemic molecular alterations rather than direct processes occurring in brain tissue. PCA of the transcriptome revealed a clear separation between patients and controls, with the first component accounting for 26.3% of the total variance. Both MD and MD + APs largely clustered together, indicating a distinct blood gene expression signature in mentally ill subjects independent of diagnosis, as BP or SSD, and medication status, although a subset of MD + AP samples overlapped with the HC cluster, indicating heterogeneity and a partial shift toward a healthy-like expression profile in some treated subjects. This finding aligns with recent studies that identified overlapping gene expression changes sharing common pathophysiological mechanisms between schizophrenia spectrum and bipolar disorders (Priyanka et al., 2025). Additionally, we observed transcriptional variability among MD + APs relative to MD, which may be attributable to the diversity of antipsychotic treatments and personalized treatment responses, both of which can influence gene expression. Antipsychotic exposure has been shown to affect the transcriptome; for example, patients on atypical APs like clozapine, olanzapine, and risperidone can exhibit more significant gene expression deviations than those unexposed (Schulmann et al., 2023).

Regarding the differentially expressed genes between MD and MD + APs compared to HC, our results show that MD and MD + APs patients have transcriptional dysregulation relative to healthy controls, with 1,373 DEGs shared between individuals on and off APs (Figure 2A). In contrast, a direct comparison of MD and MD + APs revealed only four significant DEGs (*CYRIA and TSIX, FRS2, XIST*), indicating that APs treatment has only a limited effect on the overall disease-related gene expression pattern. Therefore, we focused our analysis on the overlapping DEGs in both groups (MD, MD + APs) to better understand the fundamental pathological pathways of mental illness while minimizing the confounding effects of APs.

Functional enrichment analysis of the common DEGs revealed pathways related to immune responses, cellular homeostasis, and metabolic regulation. Our findings support the “inflammatory hypothesis” of mental illness, suggesting that psychiatric disorders have an immunological component. This hypothesis proposes that the immune system influences the development of these disorders, regardless of antipsychotic treatment (Network and pathway analysis subgroup of psychiatric genomics, 2015; Lanz et al., 2019; Priyanka et al., 2025). This is also supported by the protein–protein interaction (PPI) network, which highlights the cytokine signaling module. Disease enrichment analysis indicates that dysregulated genes are implicated in various conditions, including autoimmune diseases, infectious diseases, cancer-related processes, and neurodegenerative disorders. Additionally, a WGCNA identified a specific module (M1) that was significantly upregulated in patients. The overlap between M1 module genes and DEGs includes 966 shared genes across all datasets, indicating that this module not only reflects the overall network structure but also significantly overlaps with the most strongly dysregulated genes in individuals with mental health conditions.

Metabolic features, including BMI, waist circumference, fasting glucose, HbA1c, triglycerides, and HDL, showed no significant differences between the MD and MD + AP groups and healthy controls. This may be due to the high prevalence of metabolic syndrome components in our study population, which primarily includes Qatari and South Asian individuals, as metabolic syndrome is common among these ethnic groups—about 43% in Qatari nationals and up to 60% in South Asians. Such prevalence likely elevated these measures even in healthy controls, potentially masking the expected group differences (Syed et al., 2020). However, we observed significant differences between groups in specific cardiovascular risk markers: LDL cholesterol was higher in the MD + AP group than in the MD group, diastolic blood pressure was elevated in both the MD and MD + AP groups compared to the control group, and systolic blood pressure was significantly increased only in the MD + AP group. This aligns with the fact that antipsychotic medications can cause metabolic side effects, including adverse changes in lipid profiles (Vancampfort et al., 2015; Zhang et al., 2020). We then applied WGCNA to relate co-expression modules to metabolic traits. The resulting module–trait heatmap revealed distinct gene clusters associated with specific clinical phenotypes; however, the M1 module, which differed by diagnostic group, was not linked to any metabolic values, suggesting that it reflects pathways related to psychiatric status rather than metabolism.

We then subcategorized MD and MD + APs by diagnosis and identified DEGs in BP and BP + APs compared with HCs. To minimize the drug-related confounding factors and to focus on core disease biology, we concentrated on common DEGs shared by both BP and BP + APs. Enrichment analysis of the shared BP DEGs revealed clustering into immune-related and metabolic pathways, consistent with previous transcriptomic studies demonstrating strong immune activation in bipolar disorder. Notably, bipolar-specific signatures include upregulation of the interferon response and JAK–STAT signaling pathways (Priyanka et al., 2025). Additionally, processes related to lymphocytes and sphingolipids were enriched, consistent with earlier research linking sphingolipid metabolism to psychiatric disorders (Narayan and Thomas, 2011).

In SSD, the overlapping DEGs were enriched for core cellular and metabolic processes, including hexose catabolism, pyruvate and folate metabolism, telomere maintenance, and rRNA modification. This aligns with previous brain gene expression studies, which reported significant downregulation of glycolysis and TCA cycle genes in *postmortem* schizophrenia cortex (Middleton et al., 2002). The enrichment of telomere maintenance pathways is associated with clinical findings of shortened telomeres in patients with schizophrenia, suggesting accelerated cellular aging or chronic stress (Uzuncakmak et al., 2023). Disease enrichment of the SSD gene set includes lymphomas, benign prostatic hyperplasia, and pancreatic carcinoma. These conditions reflect immune dysregulation and oncogenic processes, suggesting that schizophrenia-related gene changes also intersect immune-oncogenic pathways.

Finally, we performed an overlap analysis to identify DEGs shared across studies. For SSD, we found six overlapping DEGs, with only one gene, SPON2, common to all examined studies. Additionally, bipolar disorder showed very limited reproducibility, with just one shared gene, OGT. This low cross-study overlap has been previously noted, suggesting that gene-expression findings in schizophrenia and other psychiatric disorders often do not replicate across different cohorts (de Jong et al., 2012). These disorders are likely highly heterogeneous, as schizophrenia is thought to consist of multiple molecular subtypes with distinct expression profiles, and bipolar disorder also shows clinical and biological diversity (Dor and Hertzberg, 2024). Therefore, adopting more standardized methodologies is essential to reduce inter-study variability.

Our study reflects systemic molecular changes in peripheral blood that may capture immune and inflammatory processes associated with psychiatric illness rather than local brain-specific pathology. While peripheral transcriptional signatures do not directly mirror molecular alterations in the central nervous system, some evidence suggests bidirectional communication between systemic immune states and brain function, as shown by the observation that the whole blood transcriptome shares significant gene-expression similarities with multiple CNS tissues (Sullivan et al., 2006). In addition, antipsychotic medications have been shown to exert immunomodulatory effects in humans, including alterations in peripheral cytokine profiles. Clinical studies and meta-analyses in patients with schizophrenia demonstrate that antipsychotic treatment is associated with reduced levels of pro-inflammatory cytokines such as IL-6 and TNF-α, shifts toward anti-inflammatory immune responses (Romeo et al., 2018; Gonzalez-Castro et al., 2024). These systemic effects on circulating white blood cells suggest that antipsychotics influence peripheral molecular pathways and may contribute to blood-based transcriptional signatures observed across medication groups.

This study has some limitations; the medication exposure was inconsistent since different APs were prescribed to MD + APs, which limits the interpretation of any drug-specific effects. Also, patients classified as antipsychotic-free were not treatment-naïve but had discontinued antipsychotic medication for at least 6 months before enrollment; thus, prior treatment effect cannot be entirely excluded. Another potential limitation is that peripheral blood findings may not accurately represent those in brain tissue. Additionally, the sample size was relatively small, which may have reduced the statistical power and reproducibility of our results. There is also an imbalance between the group sizes. Environmental factors were not controlled for, which may also affect gene-expression outcomes. Moreover, because this study was conducted in a Qatar-based cohort, the findings may be influenced by the population’s specific ethnic background, potentially limiting their generalizability to other groups.

In conclusion, by examining the common DEGs in patients with and without antipsychotic treatment, we identified a robust set of dysregulated genes and pathways that define the molecular profile of mental illness. These include immune activation, disruption of cellular homeostasis, and metabolic disturbances. Furthermore, the shared gene-expression signature we characterized may provide a preliminary framework for future investigations into blood-based biomarkers however, this require validation in larger, independent cohorts. To our knowledge, this is the first study to analyze transcriptomic changes of mental illness in a Qatar-based cohort, offering new insights into disease biology within this ethnic group.

## Data Availability

The data presented in the study are deposited in the NCBI repository, accession number PRJNA1420242.
