# Supplementary material for: Peripheral blood RNA-seq analysis in bipolar and schizophrenia spectrum disorders: modest influence of antipsychotic treatment
Source: Front Pharmacol. 2026 Feb 18;17:1745052. doi: 10.3389/fphar.2026.1745052 (PMC12957230; doi:10.3389/fphar.2026.1745052)
Supplement: Supplementary file 2 [file Supplementaryfile2.docx]

Supplementary Material

| Supplementary figure 1  MA Plots: (A) MD and HC. (B) MD+APs and HC. (C) BP off APs and HC. (D) BP on APs and HC. (E) SSD off APs and HC. (F) SSD on APs and HC. (G) all MD and MD+APs vs HC. | | |
| --- | --- | --- |
| A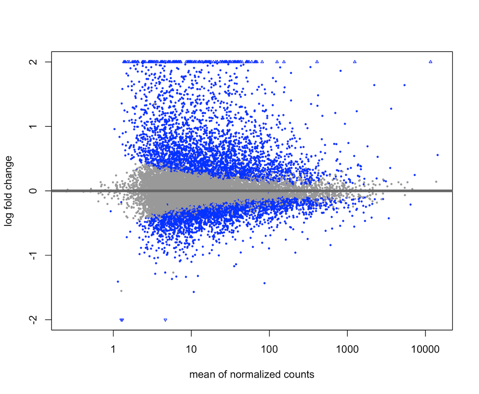 | C 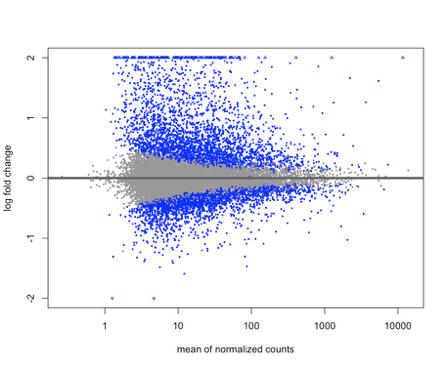 | E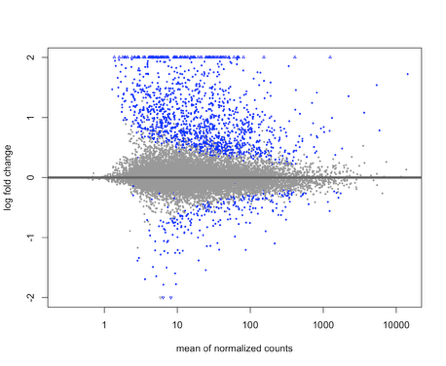 |
| B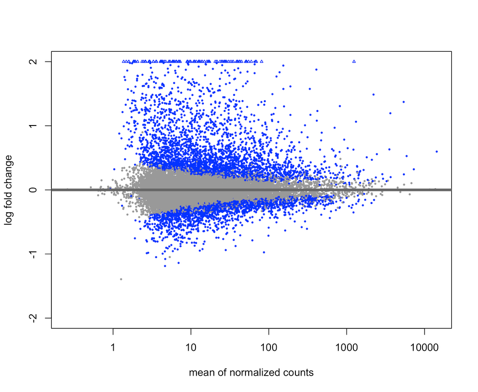 | D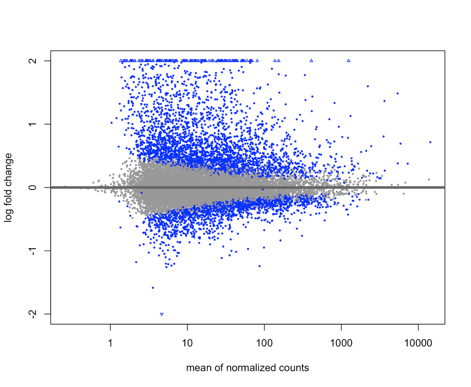 | F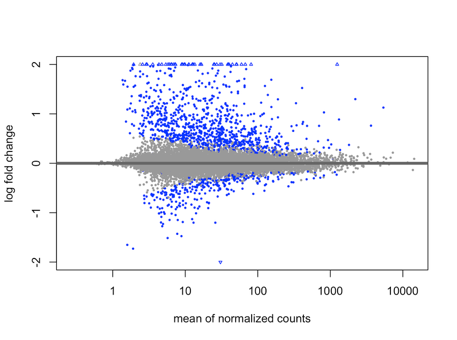 |
| G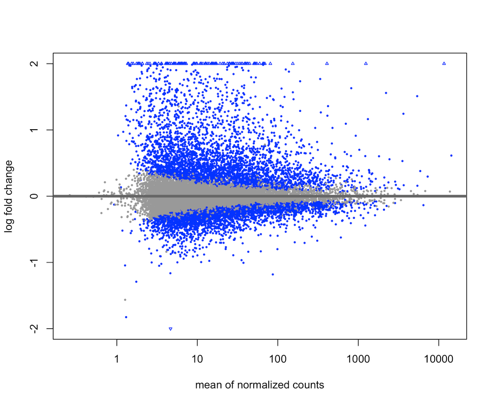 |  |  |

| Supplementary Figure 2 Principal component analysis of gene expression profiles across study subgroups  SSD: schizophrenia spectrum disease BP: Bipolar disorder, APs: antipsychotics |
| --- |
| 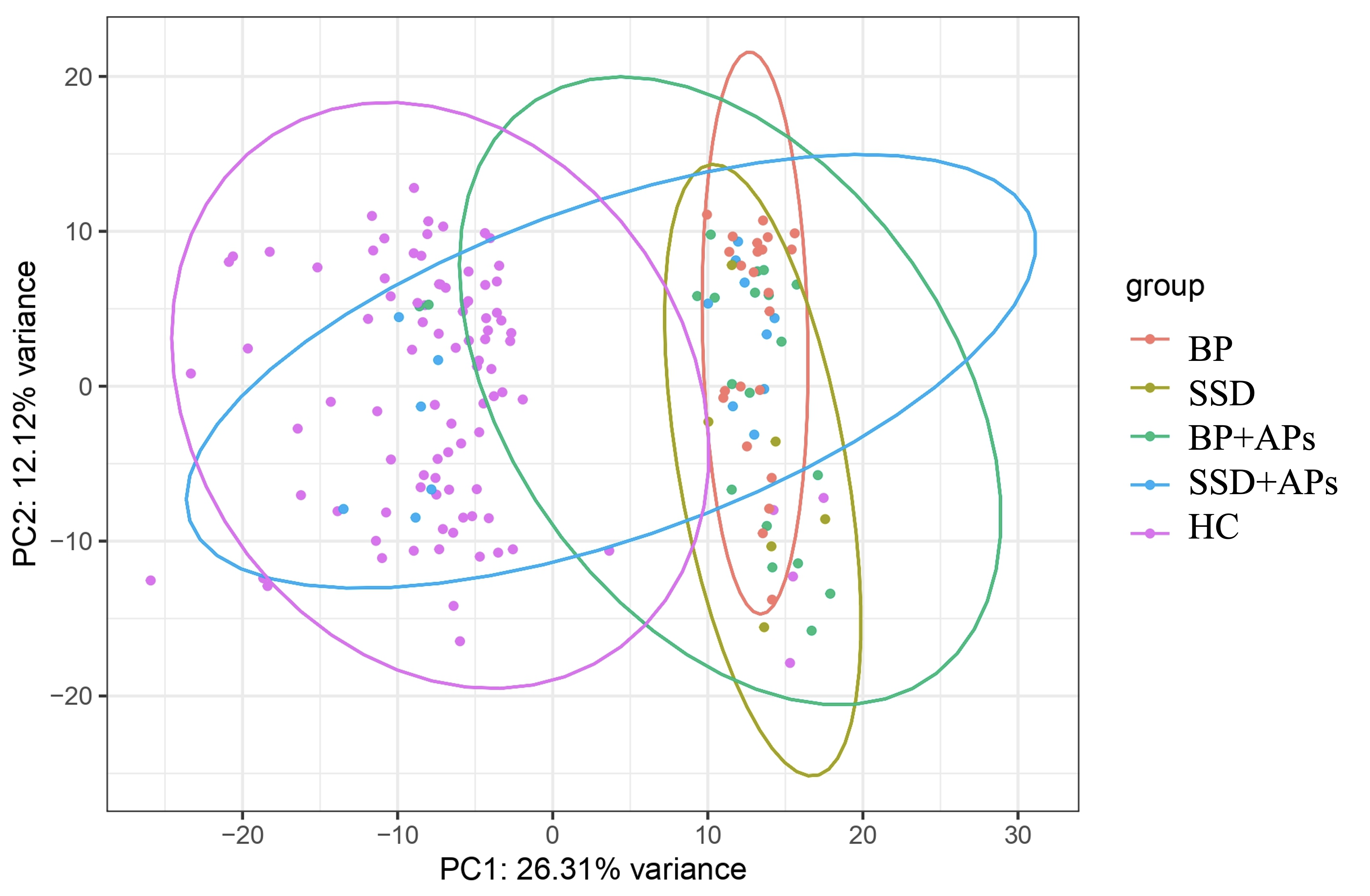 |

| Supplementary Figure 3 Heatmap enrichment of DEGs between Healthy controls and all mentally ill subjects using Metascape |
| --- |
| 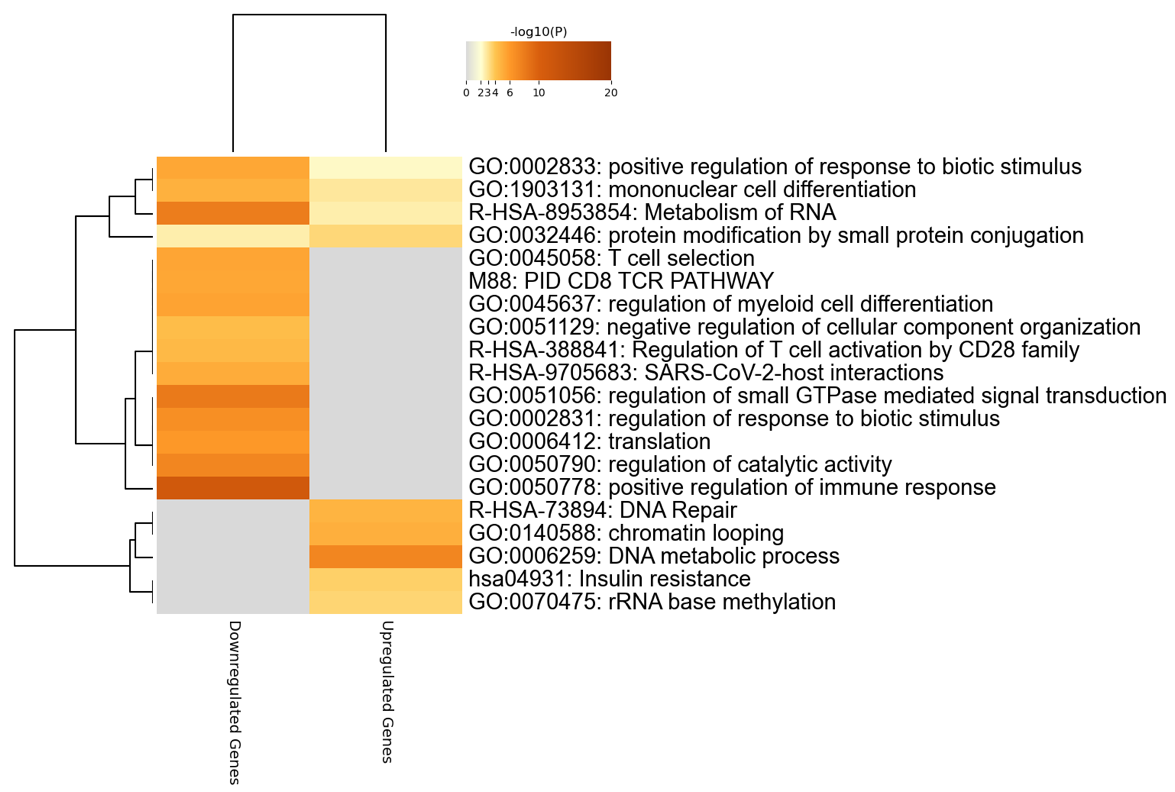 |

| Supplementary Figure 4 (A) Venn diagram of up- and down-regulated DEGs between MD and MD+APs. |
| --- |
| 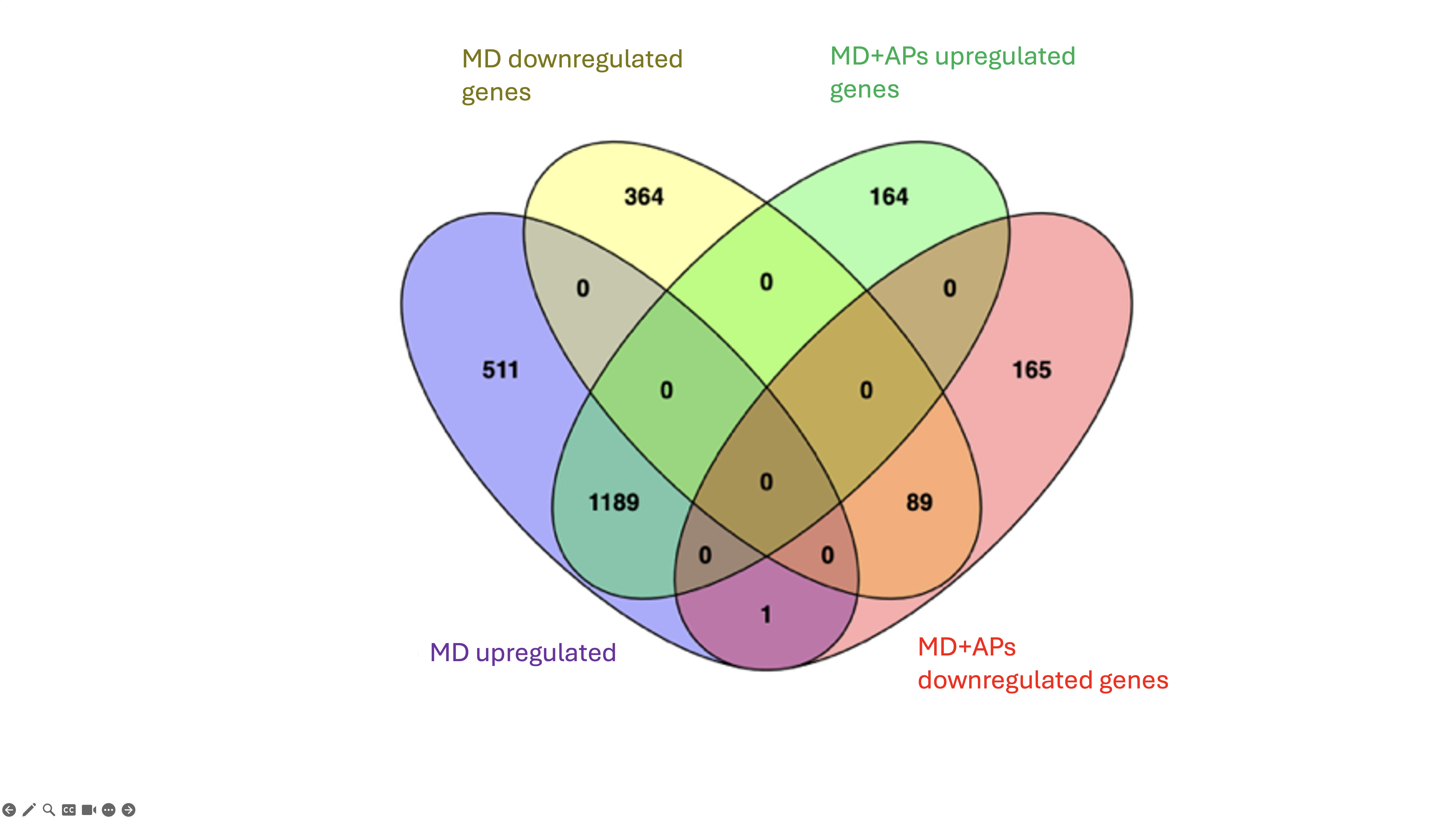 |
| Supplementary Figure 4-(B): PPI interaction network of total DEGs. The MCODE algorithm was applied to clustered enrichment ontology terms to identify densely connected proteins. Each MCODE network is assigned a unique color. |
| 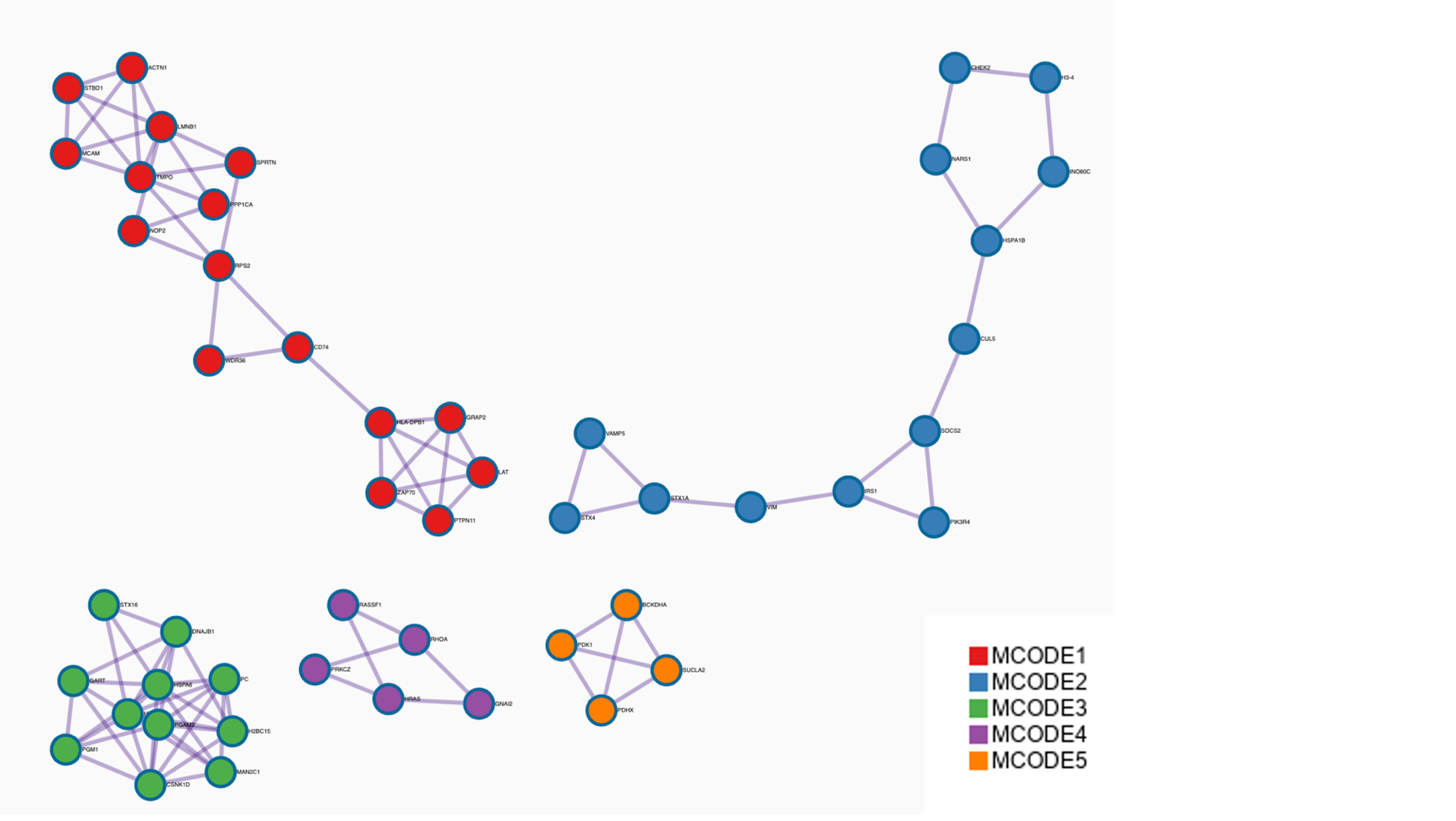 |

| Supplementary Figure 5 (A) WGCNA module M1 expression across HC, MD and MD+APs Top: heatmap of normalized expression (z-scores) of genes in module M1 across individual samples. Bottom: module eigengene values summarizing overall module expression per sample. )B( Distribution of module eigengene (ME1) values across MD, MD+APs, and HC groups shown as boxplots.” |
| --- |
| 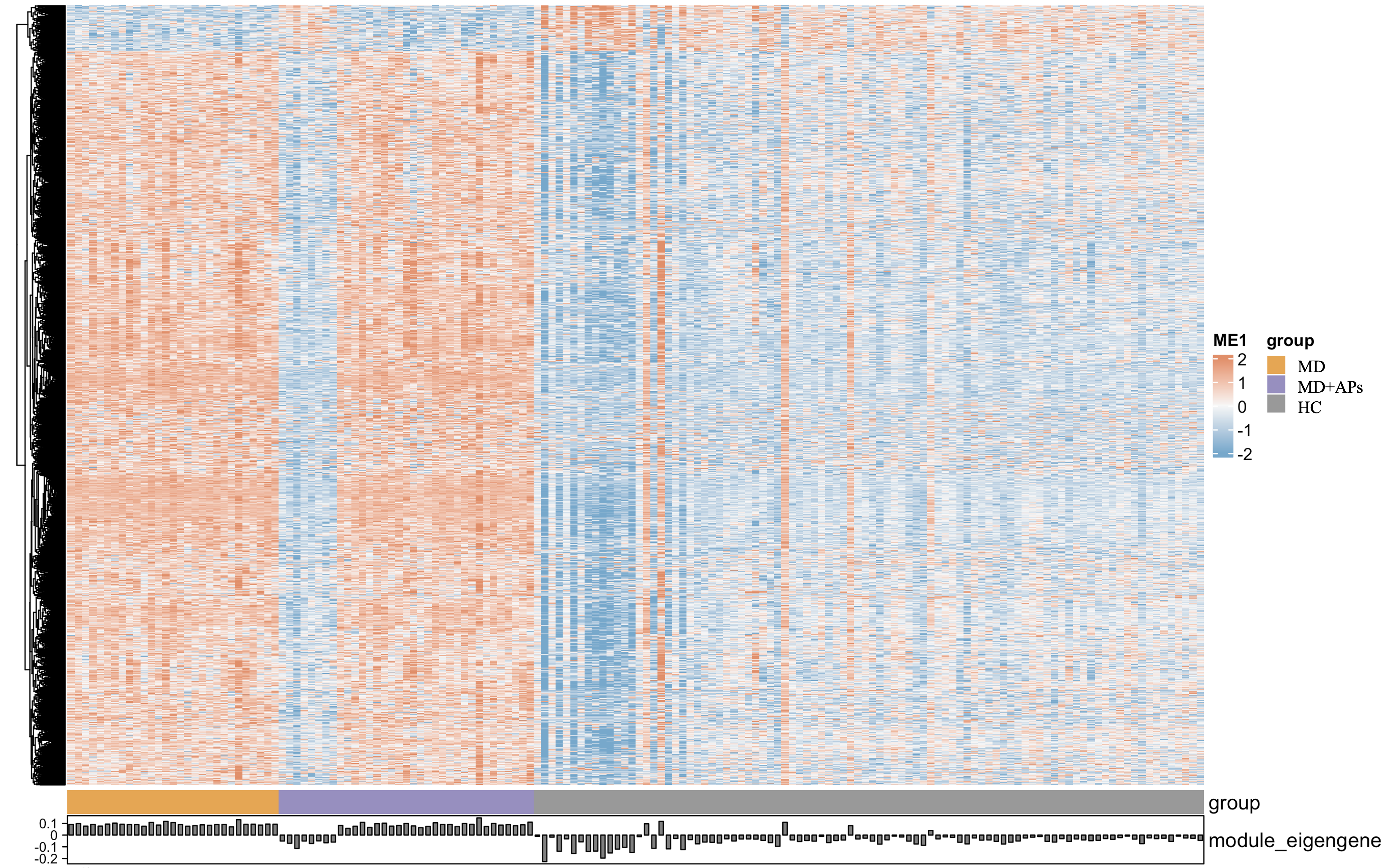 |
| 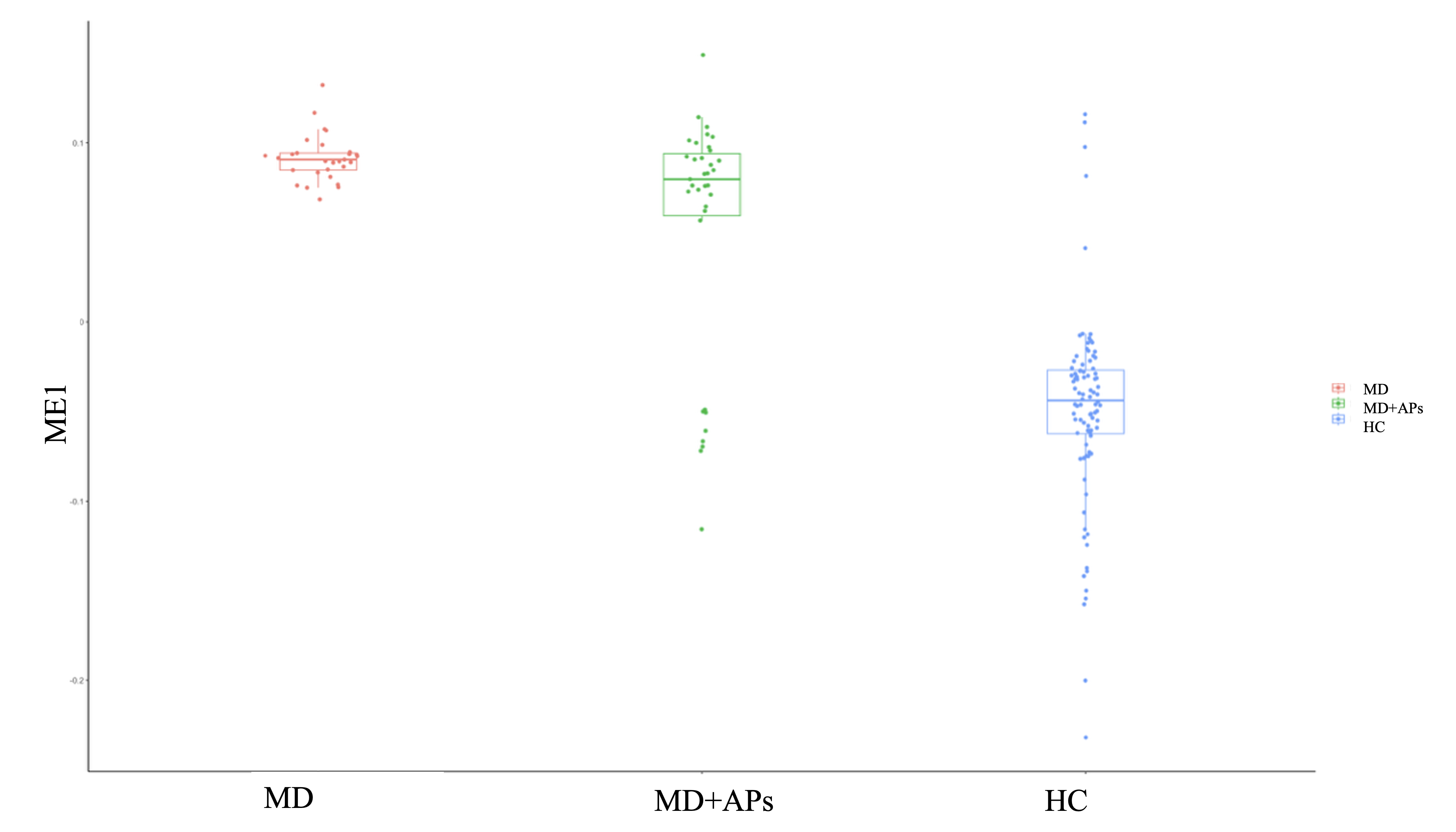 |

| Supplementary Figure 6 (A)Ven Diagram showing the common genes between the M1 modules and the MD and MD+APs differentially expressed genes. )B( Enrichment of common the M1 modules and the MD and MD+APs differentially expressed genes. )C( Summary of enrichment analysis in DisGeNET of common M1 modules and the MD and MD+APs differentially expressed genes. | |
| --- | --- |
| A  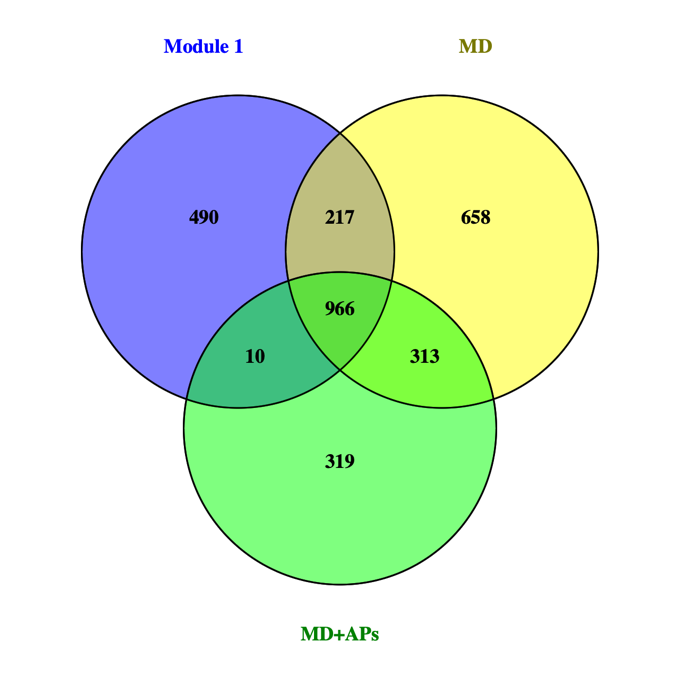 | B  C   |

| Supplementary Figure 7 Metabolic features (BMI, Waist circumference, fasting blood glucose, HbA1c and HDL) in MD , MD+APs, HC. Test used is one-way ANOVA followed by post hoc comparisons. |
| --- |
|  |

| Supplementary Figure 8 WGCNA modules association with clinical metabolic features using all samples included in the study (HC, MD, and MD+APs). Each cell displays the corresponding **p-value** for the correlation, while the color scale indicates the **direction of the correlation** (red: positive; blue: negative) |
| --- |
| 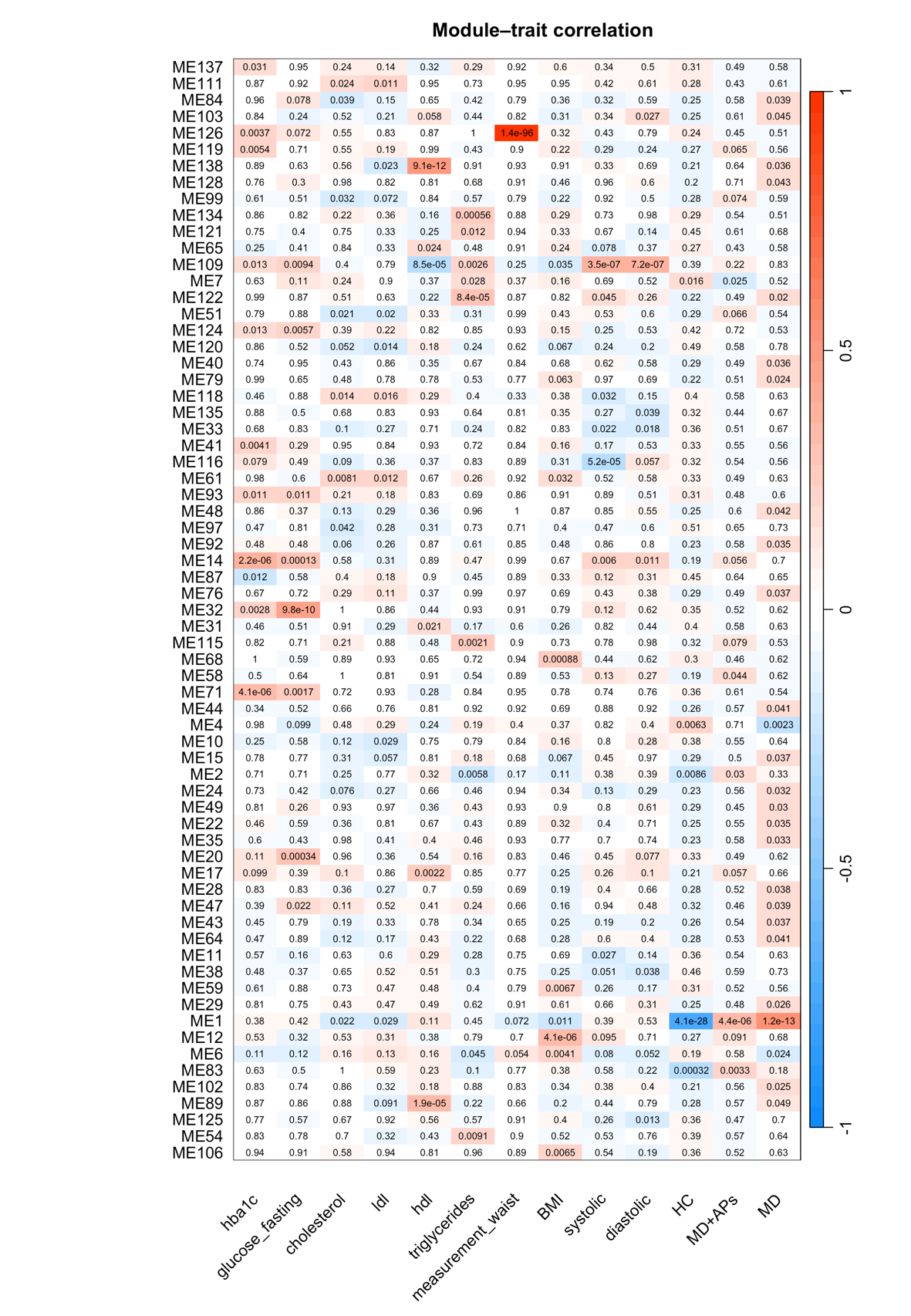 |

| Supplementary Figure 9 (A) Differentially expressed genes between BP off APs and HC. (B) Differentially expressed genes between BP on APs and HC. (C) Differentially expressed genes between SSD off APs and HC, D- Differentially expressed genes between SSD on APs and HC. | |
| --- | --- |
| a  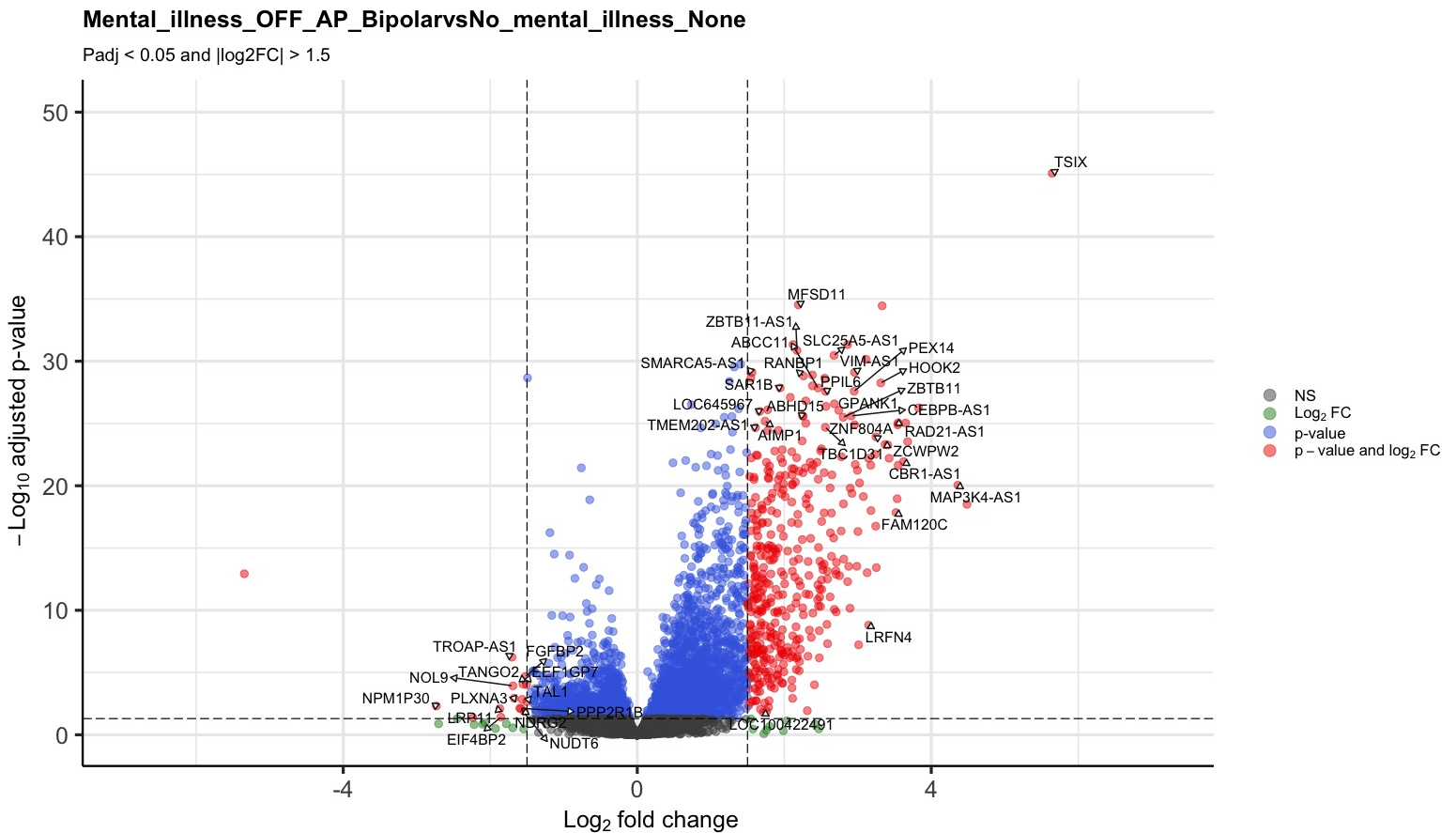 | b  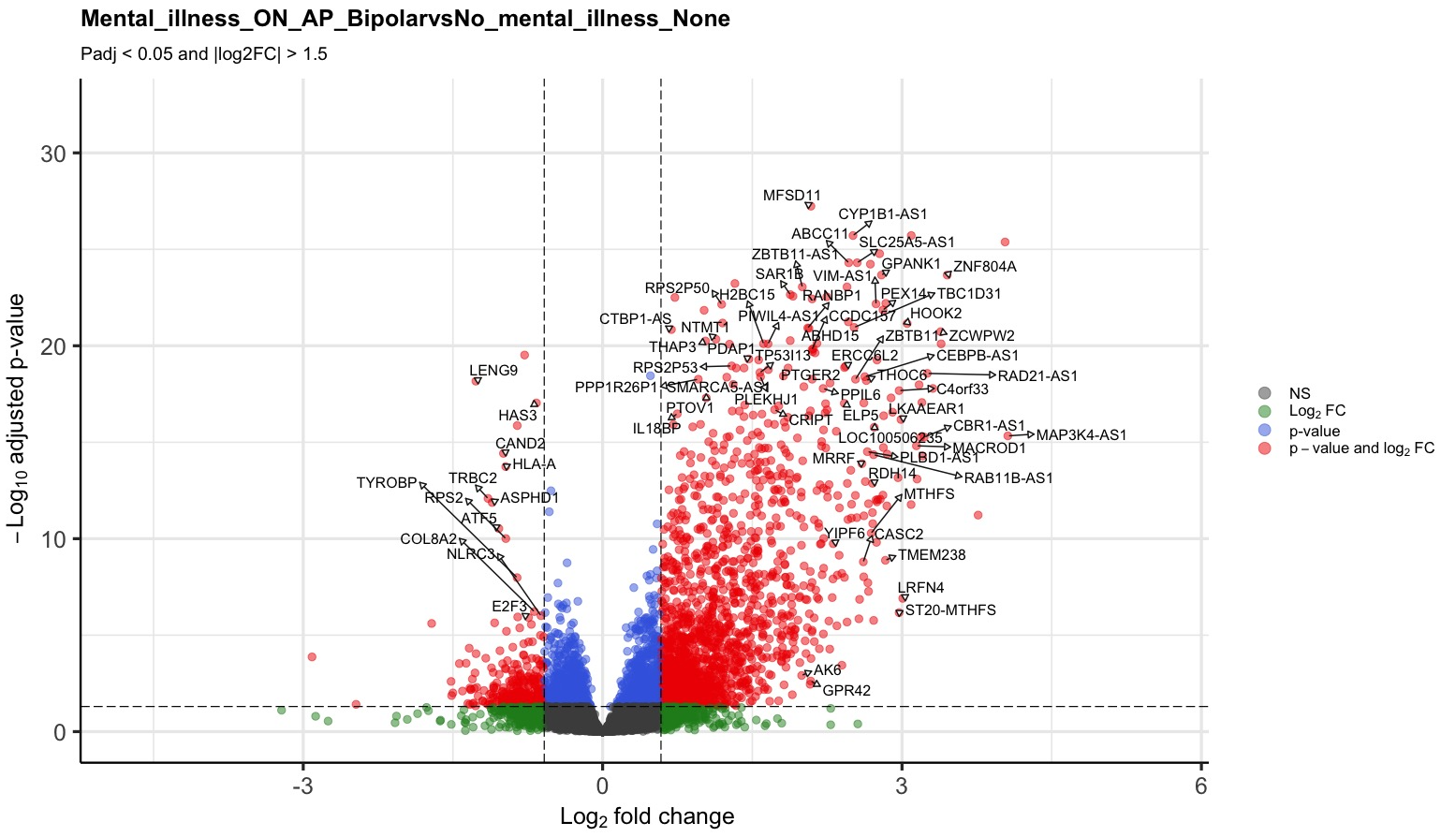 |
| c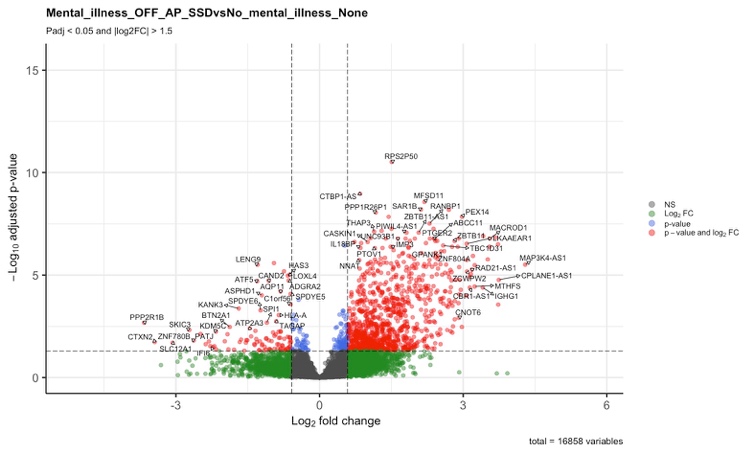 | d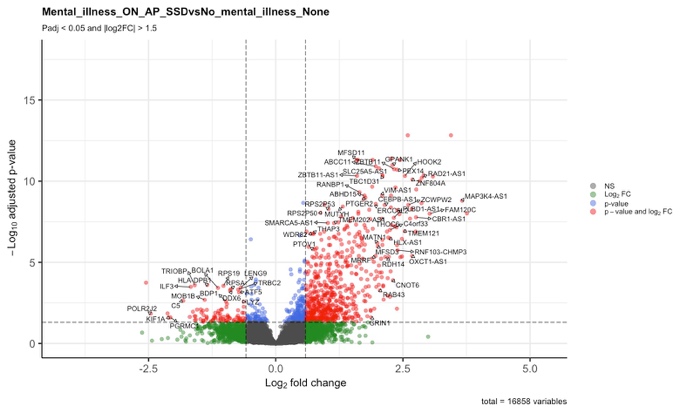 |

| Supplementary Figure 10 (A) Venn diagram of BP on and off APs, up- and down-regulated DEGs. |
| --- |
| 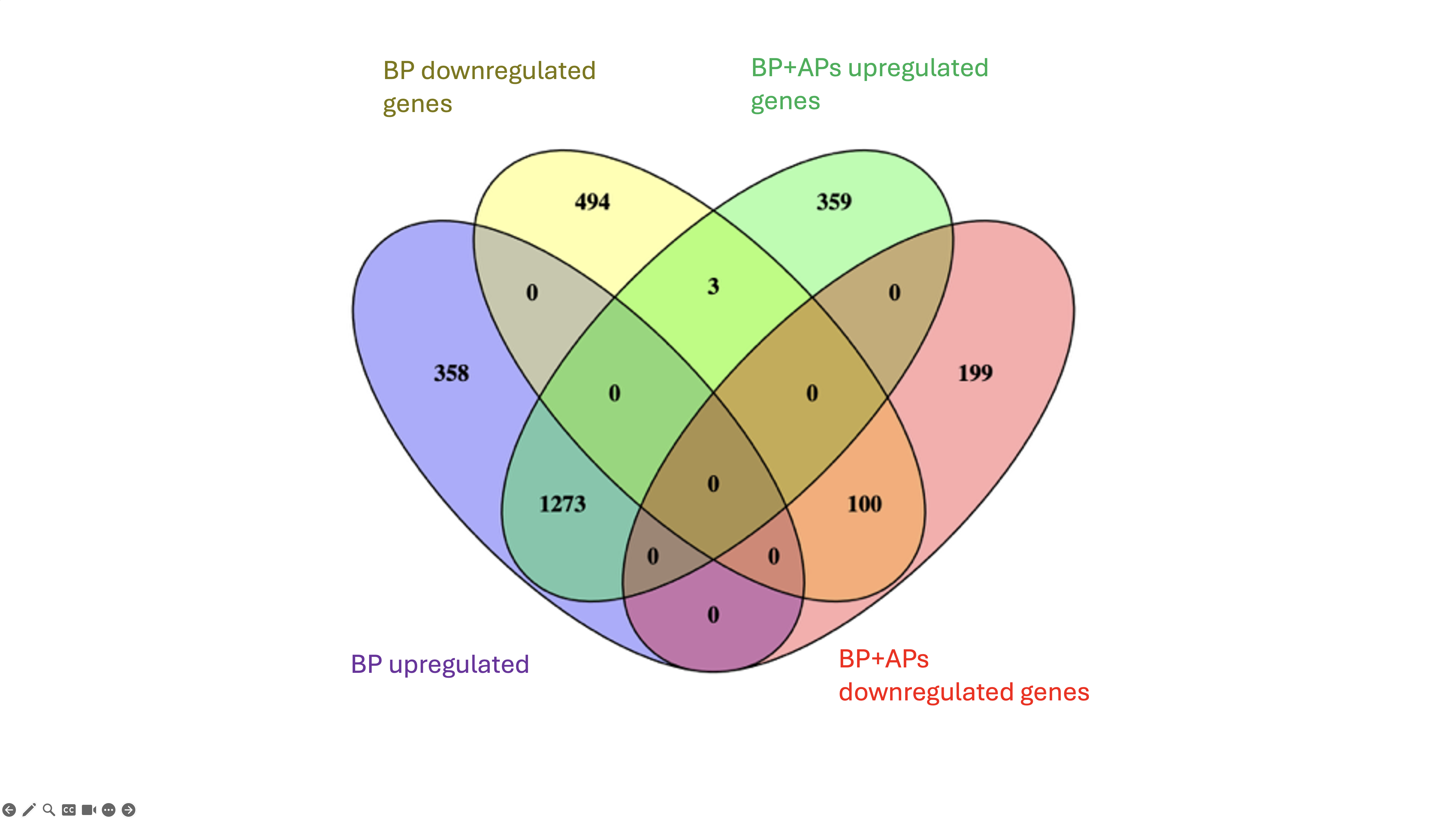 |
| Supplementary Figure 10 (B) PPI interaction network of total DEGs. The MCODE algorithm was applied to clustered enrichment ontology terms to identify densely connected proteins. Each MCODE network is assigned a unique color. |
| 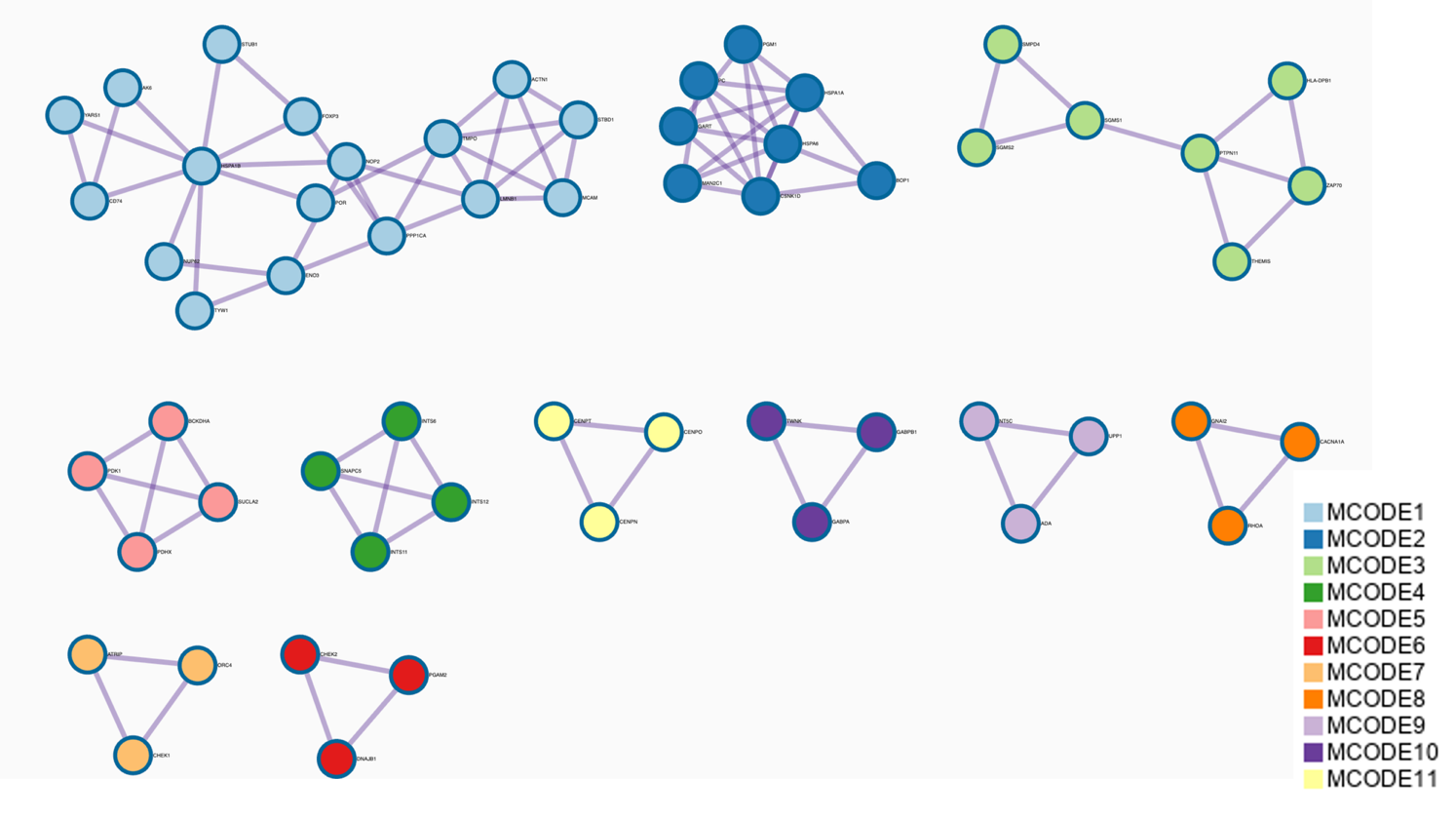 |
| Supplementary Figure 11 (A) Venn diagram of SSD on and off APs up and down-regulated DEGs. (B) Enrichment of upregulated genes between SSD off APs and SSD on APs. (C) Summary of enrichment analysis in DisGeNET of common upregulated genes between SSD off APs and SSD on APs. |
| A  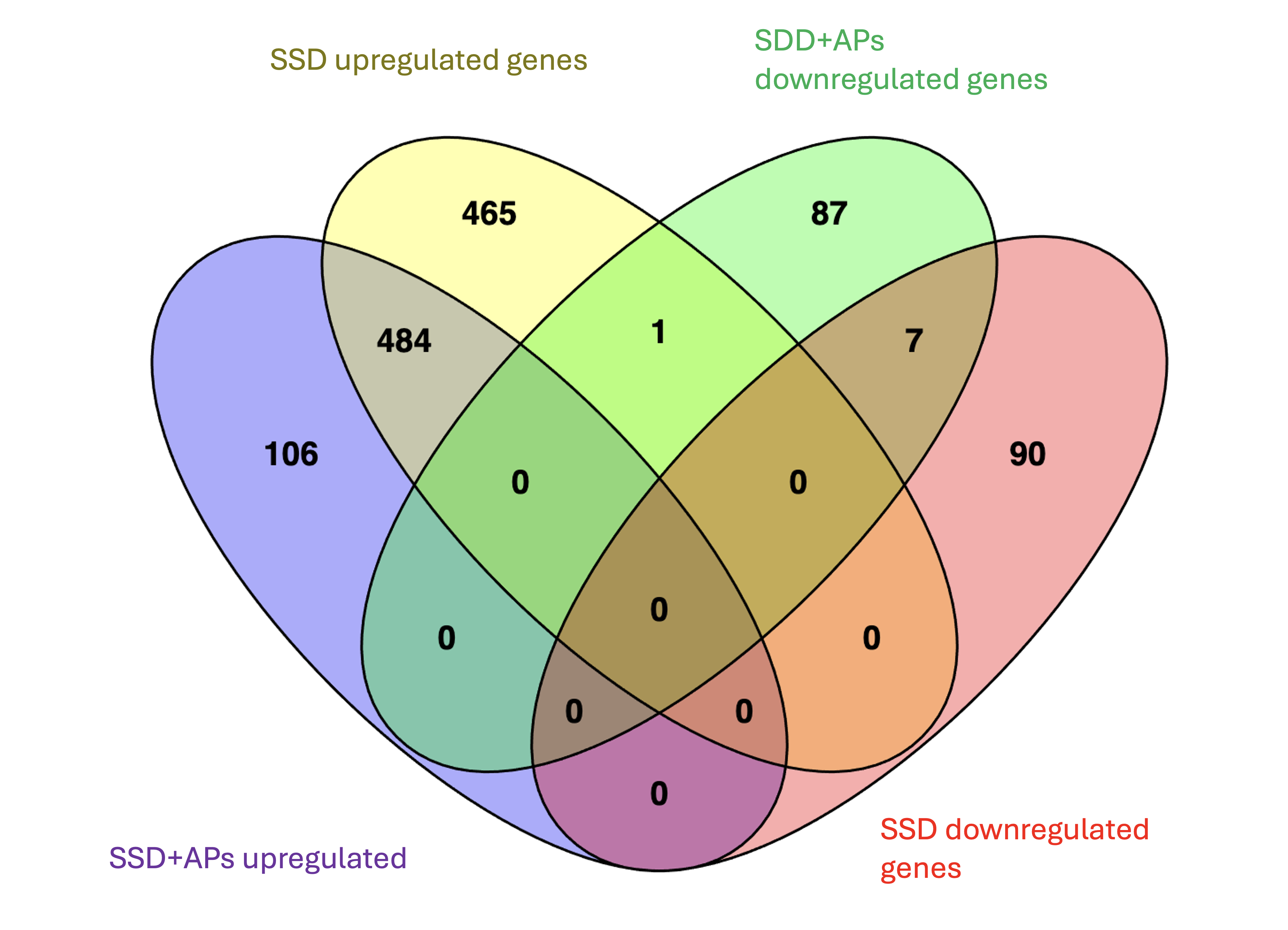 |
| B  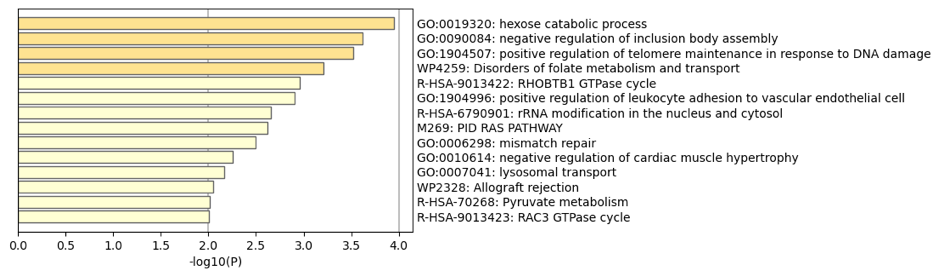 |
| C-  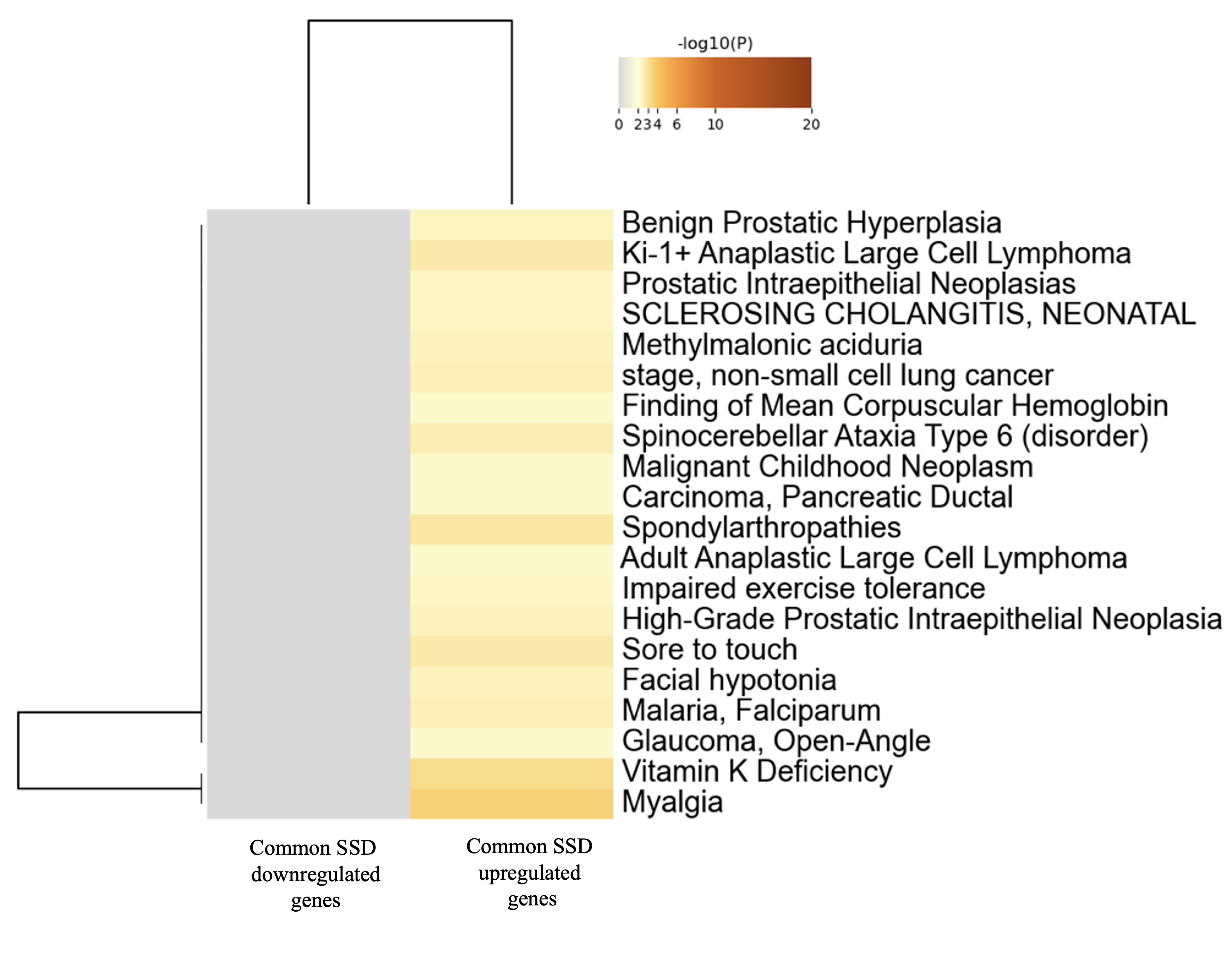 |

| Supplementary Figure 12 (A) Venn Diagram of DEGs in SSD patients on and off APs and PMID: 40015618, PMID: 38988837, and PMID: 34348681. (B) Venn Diagram of DEGs in BP patients on and off APs and PMID: 31391148, PMID: 31589133, PMID: 26241352. | |
| --- | --- |
| A  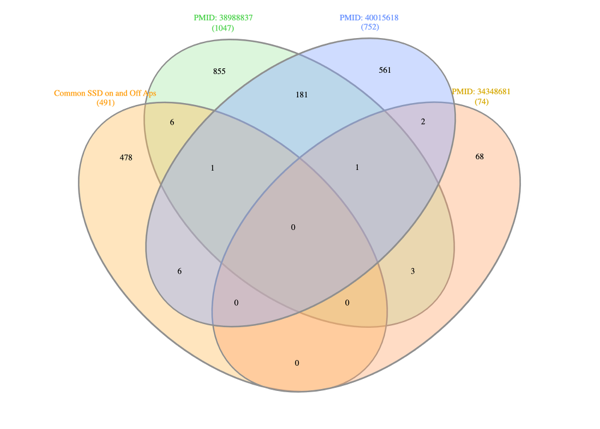 | B  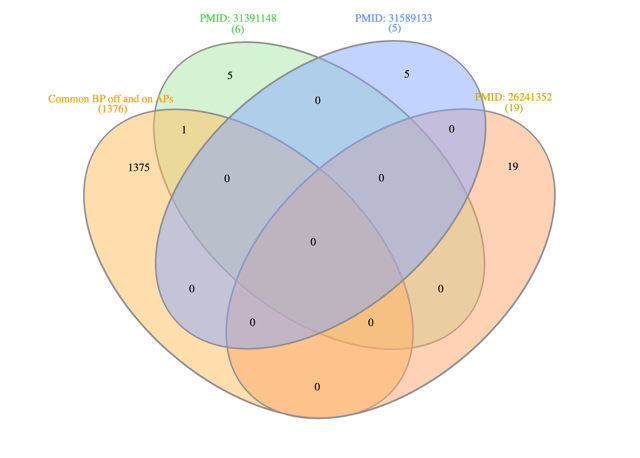 |
